# Understanding diaschisis models of attention dysfunction with rTMS

**DOI:** 10.1038/s41598-020-71692-6

**Published:** 2020-09-10

**Authors:** Javier O. Garcia, Lorella Battelli, Ela Plow, Zaira Cattaneo, Jean Vettel, Emily D. Grossman

**Affiliations:** 1 US CCDC Army Research Laboratory, 459 Mulberry Pt Rd., Aberdeen Proving Ground, MD 21005 USA; 2 University of Pennsylvania, Philadelphia, PA USA; 3 Center for Neuroscience and Cognitive Systems@UniTn, Istituto Italiano di Tecnologia, Via Bettini 31, 38068 Rovereto, TN Italy; 4 Berenson-Allen Center for Noninvasive Brain Stimulation, Department of Neurology, Beth Israel Deaconess Medical Center, Harvard Medical School, Boston, MA 02215 USA; 5 Department of Biomedical Engineering and Department of Physical Medicine and Rehabilitation, Cleveland Clinic, Cleveland, OH 44195 USA; 6 Department of Psychology, University of Milano-Bicocca, 20126 Milan, Italy; 7 IRCCS Mondino Foundation, Pavia, Italy; 8 University of California, Santa Barbara, Santa Barbara, CA USA; 9 Department of Cognitive Sciences, University of California Irvine, Irvine, CA 92697 USA

**Keywords:** Neuroscience, Cognitive neuroscience, Computational neuroscience, Sensory processing, Visual system

## Abstract

Visual attentive tracking requires a balance of excitation and inhibition across large-scale frontoparietal cortical networks. Using methods borrowed from network science, we characterize the induced changes in network dynamics following low frequency (1 Hz) repetitive transcranial magnetic stimulation (rTMS) as an inhibitory noninvasive brain stimulation protocol delivered over the intraparietal sulcus. When participants engaged in visual tracking, we observed a highly stable network configuration of six distinct communities, each with characteristic properties in node dynamics. Stimulation to parietal cortex had no significant impact on the dynamics of the parietal community, which already exhibited increased flexibility and promiscuity relative to the other communities. The impact of rTMS, however, was apparent distal from the stimulation site in lateral prefrontal cortex. rTMS temporarily induced stronger allegiance within and between nodal motifs (increased recruitment and integration) in dorsolateral and ventrolateral prefrontal cortex, which returned to baseline levels within 15 min. These findings illustrate the distributed nature by which inhibitory rTMS perturbs network communities and is preliminary evidence for downstream cortical interactions when using noninvasive brain stimulation for behavioral augmentations.

## Introduction

The effective deployment of visual attention depends on spatially competitive mechanisms distributed through frontoparietal cortical networks^1–3^. Patients with unilateral insult to these networks often suffer from hemispatial neglect, a failure to explore and orient towards contralesional space^4^. Although the severity of the hemispatial neglect typically decreases in the months following stroke onset, many of these individuals are left with permanent visual “extinction” in which events in the contralesional visual field are ignored when they occur simultaneous with salient events in the ipsilesional field^5,6^. Despite the active study of neural mechanisms supporting visuospatial attention, the means by which maladaptive chronic deficits in visual extinction persist are still poorly understood.

Neglect models recognize that localized insult to the attention network have cascading effects that propagate throughout the attention network^1,5^. Interhemispheric competition is a key mechanism in these models, in which mutual inhibition is disrupted between transcallosally-connected homotopic circuits, resulting in an upregulation of excitation in the contralesional tissue that is otherwise healthy^7–12^. In addition, the fontoparietal attention network is strongly connected within-hemisphere via association fibers, the structural and functional connectivity of which are both essential for effective deployment of visuospatial attention^13,14^. Thus, there exists multiple possible mechanisms by which *diaschisis*, or the downstream destabilization of functionally connected circuits, may promote and sustain the maladaptive functional organization of attention systems observed in chronic extinction.

The goal of this study is to characterize how the targeted insult within the attention networks may restructure mesoscale network architecture in the brain. We hypothesize that disrupting a single node of the attention network has the potential to shift the dynamics of network organization downstream from the stimulation site, mediated by functional connected circuits. This is based, in part, on the observation that acute and localized insult has potential to alter the hodological, or network, spreading of dysfunction^15–17^.

In this study, we induced disruption using noninvasive offline, repetitive transcranial magnetic stimulation (rTMS), delivered to the intraparietal sulcus (IPS), a key posterior node of the dorsal attention system^18–20^. Repetitive TMS is a particularly effective tool for modulating large-scale brain networks, including downstream from the stimulation site, by employing a propagation of pulses that travel through functionally connected systems^18, 21–26^. Moreover, rTMS is a neuromodulatory tool that induces transient metaplastic changes in cortical circuits that are sustained well beyond the time of stimulation^27–30^. The extended action of rTMS makes it well-suited for pairing with functional magnetic resonant imaging (fMRI) using a “condition-and-map” approach by which the durable impacts of neuromodulation on the dynamics of functionally connected circuits can be evaluated^31^.

To evaluate the non-localized impact of inhibitory rTMS, we use network science tools by which large-scale functional brain systems may be described, including how they dynamically organize into interconnected communities and are perturbed by interventions^32,33^. Using these tools, researchers have identified an architecture in healthy brains that reflects a balance of segregation and integration in functionally specialized systems that is flexible to task demands^34^. Localized damage from stroke is linked to poor segregation in the lesioned hemisphere that spreads to the healthy (i.e., unlesioned) hemisphere^16,35–37^. And although modular community structure provides a level of robustness that protects network architecture against disruption induced by insult^38,39^, localized lesions to the highly integrative areas may in fact promote disease spreading^39^ and encourage more severe behavioral impairments^17^.

In this study, we focus on the community dynamics that evaluate time-varying interactions of nodes and network configurations. These metrics quantify the movements of individual nodes among network community affiliations (flexibility, cohesion, promiscuity), and quantify the dynamic deviations of nodal motifs from a standard model (recruitment and integration). These metrics, in particular, have been effective for identifying potential biomarkers of dysfunction in higher cognitive systems^40^ and have been proposed as potential indicators of positive outcomes in the context of rehabilitation after injury^41^. For these reasons, we hypothesize that the network dynamics may be informative for describing the destabilization of brain systems induced by neuromodulation.

Collectively, our results reveal rTMS transiently alters the integrative properties of brain regions distal from the stimulation site, particularly in prefrontal cortex. In these healthy participants, network dynamics restabilize to their archetypal (or stable) state within a time interval that approximates stimulation time. Our findings are evidence that the propagation of noninvasive brain stimulation may capitalize on the flexible dimensions of neural community architecture, with localized inhibition increasing network dynamics distal from the stimulation site.

## Results

This study proceeded as a “condition-and-map” paradigm, in which participants received 15 min of 1 Hz inhibitory active stimulation (or sham, collected in a separate session on a separate day) over the left intraparietal sulcus. rTMS followed immediately, within 5 min, by the initiation of fMRI. During the brain imaging measures, participants engaged in bilateral visual tracking designed to elicit competitive interactions within the cortical attention system. Participants engaged in this task for three sequential 12-min scans. The analyses below reflect the characterization of network dynamics over the course of those 36 min following stimulation.

### Community structure

We first identified the community structure in networks over the course of the task using *dynamic community detection*^42,43^, which is an algorithm to distill complex connectivity matrices (e.g., coherence matrices) into series of coarse clusters of networks across time (see Dynamic community detection in “Materials and methods” section for specifics). In brief, this method optimizes the modularity function Q such that network assignments reflect a community organization that is the most different from a null model, composed of shuffled weighted edges derived from the observed connectivity estimates. After derivation of these temporally evolving communities, we may then characterize these network dynamics with metrics that describe specific community changes across time. We first use this method, however, to identify the consensus community structure in the sham condition, to reflect the most common community organization across time and space.

**Figure 1:**
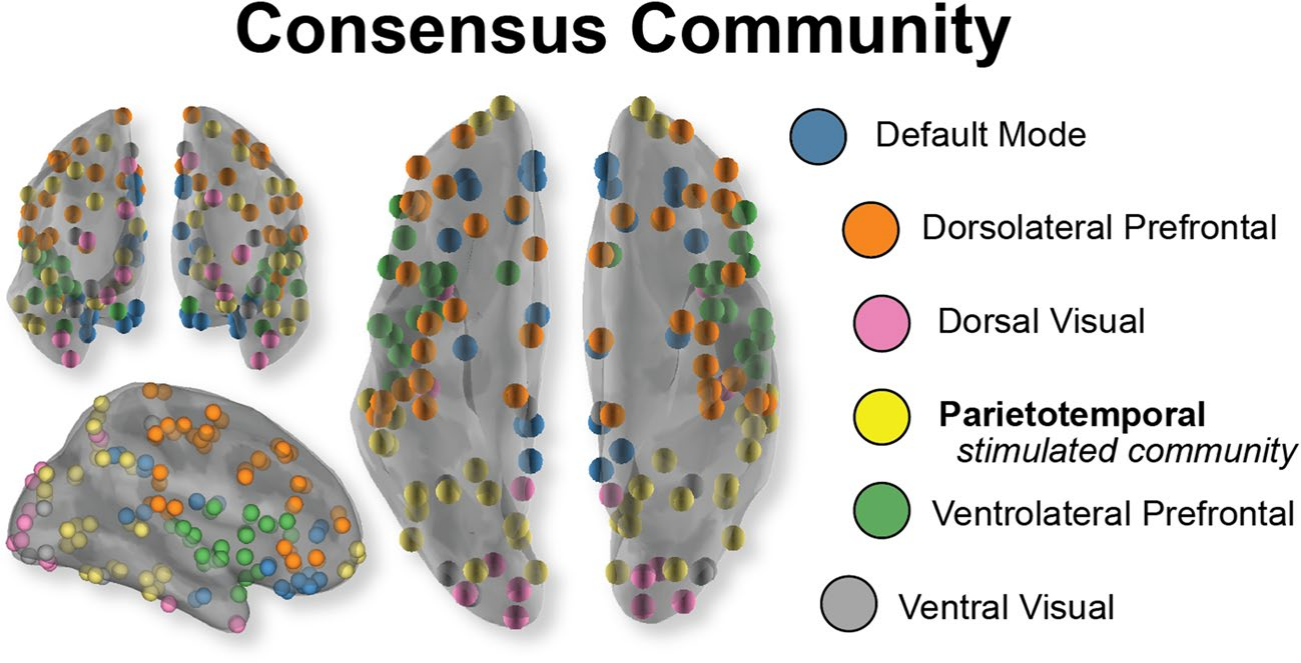
Community architecture during visual tracking, as assessed in the sham condition. Region centroids are represented as orbs plotted on a semi-transparent inflated brain. Colors indicate the consensus community structure, or the most common network architecture found across time and participants. Results indicate that on average this community structure is most common across time and participants with, on average, an 84.6% similarity in partitioning of this community structure to the others across participants and time.

As shown in Fig. 1, cortical coherence dynamics are best characterized by six communities: a dorsal visual community (pink); a ventral visual community (gray); a parietotemporal community (yellow); a community capturing nodes of the default mode network (blue); a ventrolateral prefrontal community (green); and dorsolateral prefrontal community (orange).

### Community dynamics are robust to inhibitory rTMS

**Figure 2:**
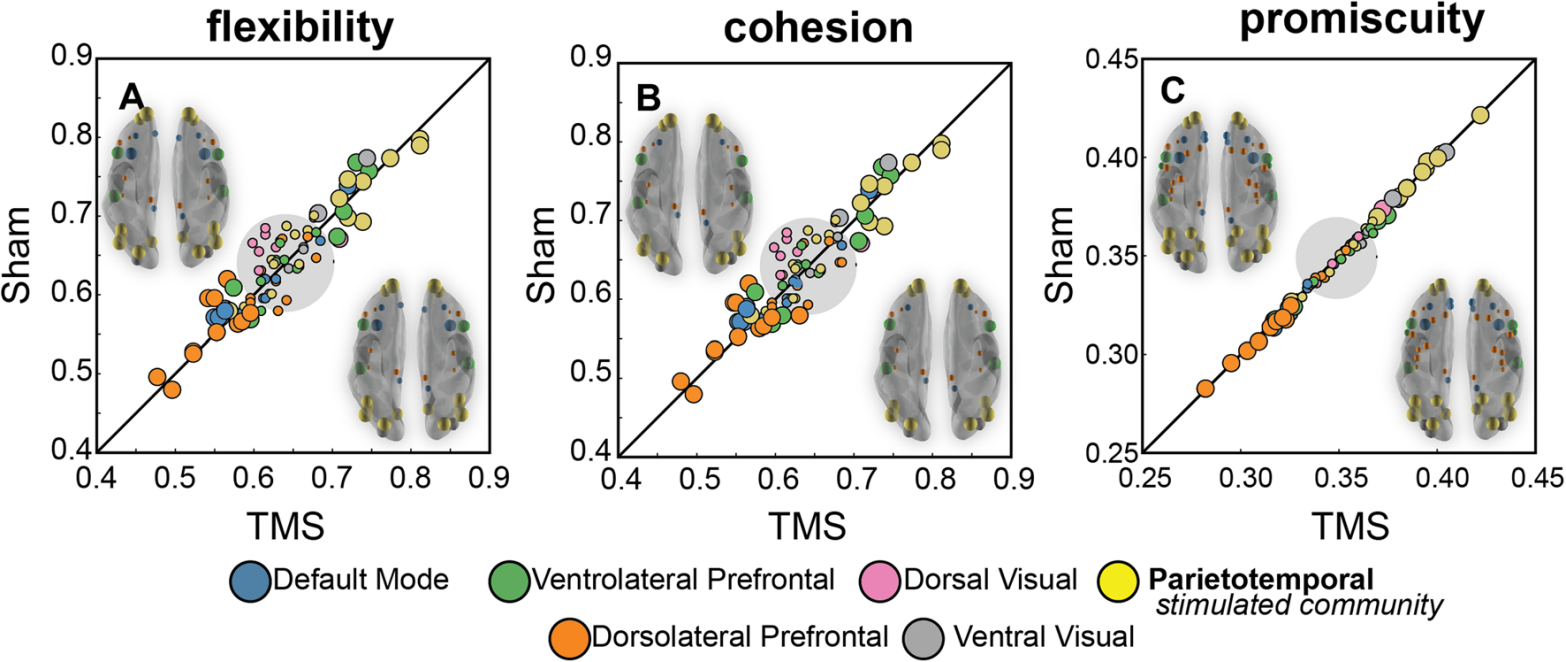
Nodal community affiliation metrics in the rTMS and sham conditions. (**A**–**C**) Average metric for each node across participants for each of the estimated individual nodal metrics, plotted with the corresponding color of the representative community as indicated by the legend. Circular gray region denotes metrics scores expected by chance (see Population distributions of node metrics in “Statistical significance” section for calculation specifics). Those scores exceeding 95% of the estimated null distribution are shown as larger tokens exceeding the null region. Semi-transparent inflated mesh brains inlayed in the upper left and lower right of each panel illustrate the spatial location of the nodes with metrics more extreme than expected by chance, with the size of the node scaled to the relative strength of the metric score. Images in the upper left of each panel show associated metric score as assessed in the sham condition and those in the bottom right are from the TMS condition.

A comparison of the individual node metrics in the rTMS and sham conditions revealed that neuromodulation had little impact on the overall community dynamics when assessed over the entire 36 min following stimulation (Fig. 2). Node flexibility (movement between communities), cohesion (the movement of network motifs), and promiscuity (dispersion of movement across communities) did not differ statistically when assessed for the rTMS and sham conditions via a series of paired sample t-tests. When comparing sham to rTMS for each metric, no node survived FDR correction for multiple comparisons (q < 0.05)^44^.

Indeed, a correlation between the means across subjects of these metrics showed that compared to the sham condition, rTMS metrics explained 84.1% (r = 0.92,  *p* < 0.001), 84.2% (r = 0.92,  *p* < 0.001), and 99.6% (r = 0.99,  *p* < 0.001) variability in the metrics for the sham condition for flexibility, cohesion, and promiscuity, respectively. These results show that local dynamics are largely robust to the perturbations introduced by inhibitory rTMS.

### Evaluating community dynamics

Although node dynamics were remarkably stable when pooled over 36 min of scanning, three communities contained subsets of nodes that were among those that exhibited extreme changes in community affiliation. These communities were characterized by nodes with dynamics that deviated significantly from what would be expected by chance (when assessed against a bootstrapped distribution of sample means, as shown as the gray shaded region in Fig. 2; see Population distributions of node metrics in the Statistical Significance segment in “Materials and methods” section). These nodes were largely within the parietotemporal community (PT; yellow), which included the stimulation site; the community organized around nodes in ventrolateral prefrontal cortex (VLPFC; green); and nodes aligned in a community distributed regions through dorsolateral prefrontal cortex (DLPFC; orange).

Nodes in left parietal cortex, which were targeted by the rTMS, were affiliated with a community characterized by high flexibility, cohesion, and promiscuity of the nodes. In other words, parietal brain regions changed community affiliation more than the population as a whole (high flexibility), with the new community affiliations tending to occur jointly with other nodes from within the PT community (high cohesion). These new community affiliations were also distributed across many networks (high promiscuity). Together, these characteristics illustrate the potential for nodes within the PT community to dynamically reorganize with other communities to promote large-scale integration across brain systems.

In contrast, nodes in the dorsolateral prefrontal cortex communities (shown in orange) revealed largely the opposite pattern of community dynamics. These nodes were less likely to change community affiliation (lower flexibility), and when nodes in this network altered their community allegiance they tended to do so in isolation (low cohesion) and to relatively few other communities (low promiscuity).

Although more diverse than the PT community, nodes in the ventrolateral prefrontal cortex demonstrated a similar pattern, with nodes cohesively changing community affiliation more frequently than expected by chance. The ventrolateral prefrontal cortex nodes, however, shifted affiliations across communities (promiscuity) consistent with that expected from a system of this modularity.

### Inhibitory rTMS changes to community allegiance

We next characterized the rapid network reconfigurations induced by rTMS by evaluating the node dynamics within shorter sliding windows of time. Functionally-driven architectures evaluated over the entire scan session reflect a stationary snapshot of what is an inherently dynamic system, with rapid reconfigurations dependent on specific task contexts and constrained by the underlying structure of the network^45,46^. This time-dependent analysis considers both the dynamics of individual nodes (as above) and a quantitative measure of deviations of nodal motifs from a standard, reference community model. The reference system in this analysis is the consensus community structure as observed in the sham condition, which reflects the functionally-driven interactions without the rTMS intervention.

Recruitment is a measure of dynamic deviations from a model system, quantifying the extent to which nodes of a particular system cohere in the same community over time. High recruitment indicates sustained and stable movements in community affiliation between nodes of the same system, and low recruitment indicating a decrease in allegiance following rTMS. In contrast, the integrative properties of a system characterize the extent to which nodes of a particular system have high allegiance to nodes of other systems. High integration is indicative of a node that shares the same affiliation frequently with nodes from other systems (i.e., high inter-system allegiance), either in a sustained or flexible manner, whereas low integration is characteristic of a node that is weakly allied with nodes in other functional systems.

**Figure 3:**
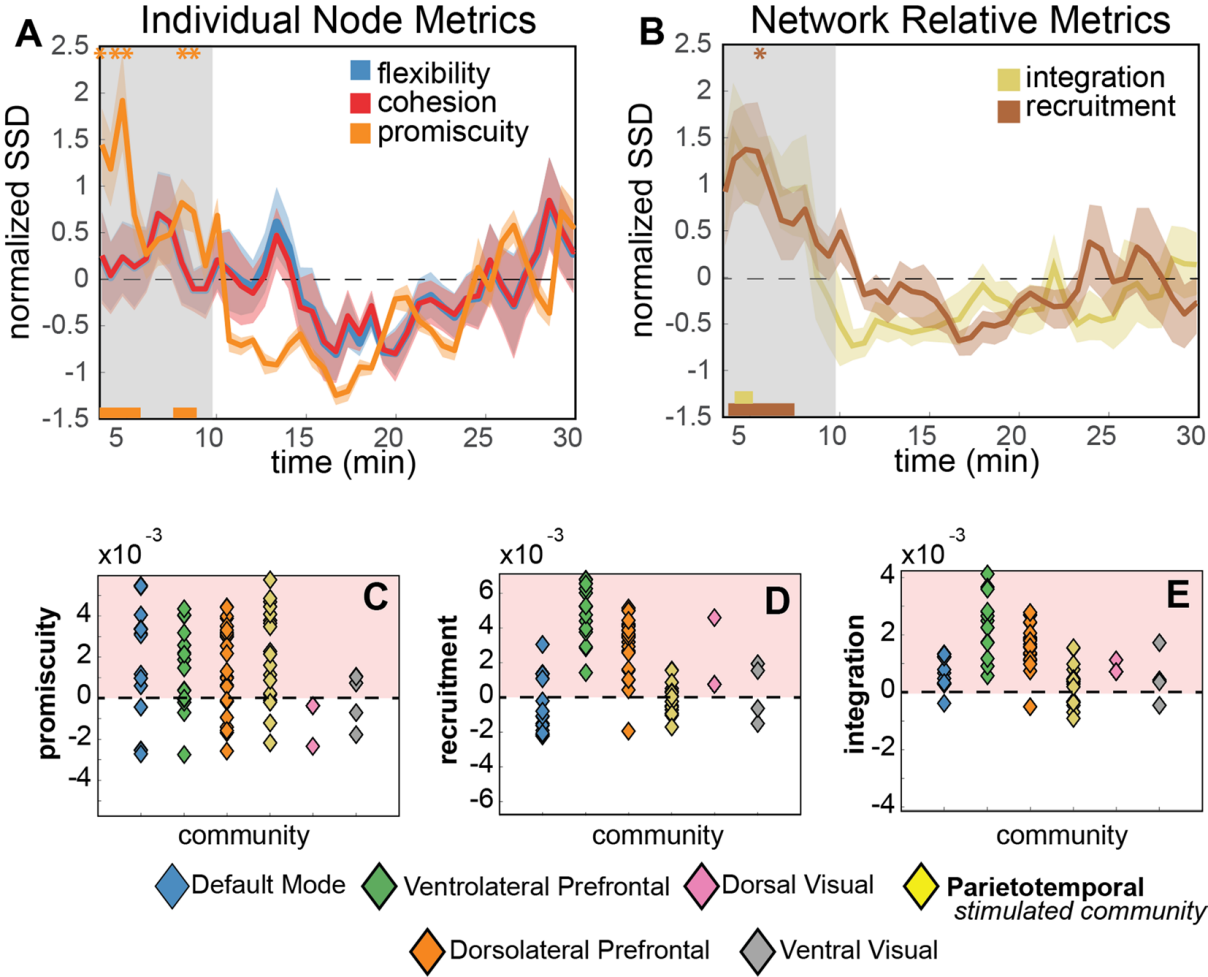
rTMS-related nodal and motif dynamics. (**A**) Time-dependent changes in node metrics (flexibility, cohesion, promiscuity) following rTMS. Normalized SSD is the sum of squared differences (SSD) between sham and rTMS conditions, aggregated over all community nodes. Error bars indicate standard error of the between-subject means, computed from individual participant z-scored SSD timeseries. Metrics are estimated in approximately 40 s windows for concatenated 12 min scans. The shaded region isolates the first 10 min following stimulation during which the metrics most strongly deviate from the community structure as evaluated during Sham baseline. Bars along the bottom axis represent significant time points (uncorrected) from as single sample t-test, with color indicating metric (e.g. orange = promiscuity). Asterisks (*) at the top of the figure indicate significant time points following an FDR correction for multiple comparisons (i.e. time point comparisons). (**B**) Recruitment (stability vs flexibility) and integration (connectedness vs isolation) coefficients over time. (**C**–**E**) Promiscuity, recruitment and integration computed during the first 10 min following rTMS (vs sham), shown for each node and organized by community.

In the first 10 min after stimulation, an average of 62% of the nodes increased their promiscuity as compared to the standard (sham) network, 86% of nodes increased their recruitment properties, and 72% of nodes increased their integration characteristics. Statistical single-sample t-tests of the mean normalized SSD (Fig. 3) identified temporal windows within the first 10 min following stimulation in which node promiscuity across the entire brain significantly increased after stimulation as compared to sham (at 3.3 min: t(6) = 3.9, *p* = 0.008); 4.0 min: t(6) = 3.0, *p* = 0.024; 4.7 min: t(6) = 3.6, *p* = 0.011; 8.0 min: t(6) = 3.1, *p* = 0.022; and 8.7 min: t(6) = 3.4, *p* = 0.014). No temporal intervals survived multiple comparison correction when comparing flexibility and cohesion after the active stimulation and sham conditions.

When corrected for multiple comparisons, system recruitment increased significantly as compared to sham approximately 6.0 min after stimulation (t(6) = 6.3, *p* = 0.001). These changes in the network dynamics returned to a stabilized state over a duration roughly equivalent to the duration of stimulation, which is consistent with the expected duration of impact as assessed behaviorally^20,47^.

**Figure 4:**
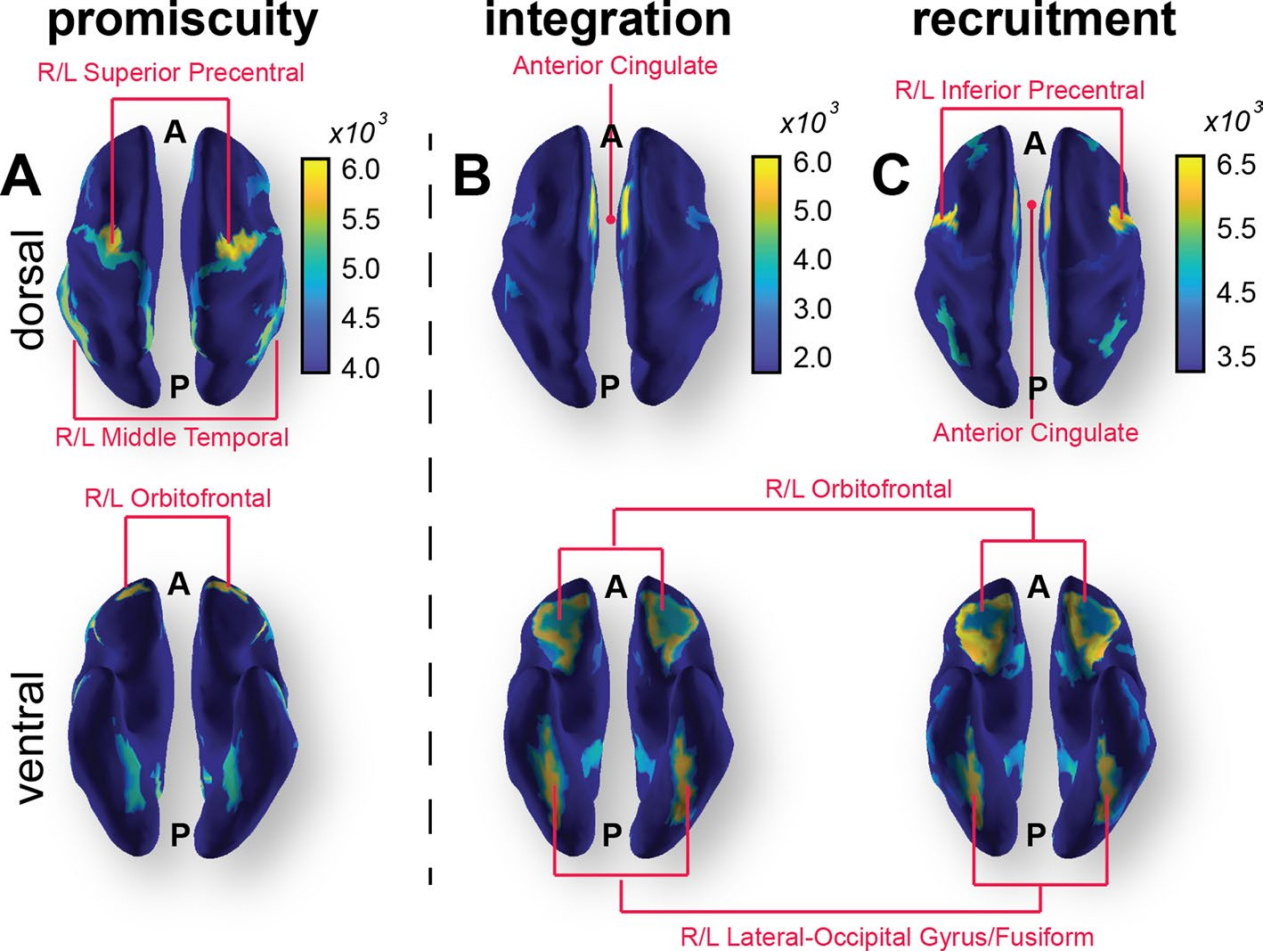
Changes in promiscuity and allegiance metrics immediately following rTMS. Color maps correspond to the top 20% regional increases of these metrics within 10 min of the delivery of rTMS. Top row: dorsal view. Bottom row: ventral view. (**A**) Nodes with greatest increase in promiscuity included bilateral dorsal precentral cortex, orbitofrontal and middle temporal cortices. (**B**,**C**) Nodes with the highest integration (left) and recruitment coefficients (right) include anterior cingulate, bilateral orbitofrontal and fusiform, and precentral sulcus (recruitment only).

The largest transient increases in recruitment and integration of nodal motifs following rTMS were predominantly within the ventrolateral and dorsolateral prefrontal cortex communities: bilateral orbitofrontal cortices, the anterior cingulate and the fusiform (Fig. 4). These nodes exhibited increased allegiance within their own and to other communities. Supplementary motor areas of the precentral cortex also exhibited high recruitment and promiscuity following rTMS, indicating sustained allegiance within its own system with increased connectedness of individual nodes to other communities. Unexpectedly, rTMS did not substantially impact the allegiance of nodes directly under the stimulation site, evidenced by the relatively small change in integration and recruitment.

### Integrative properties of the stimulation site

**Figure 5:**
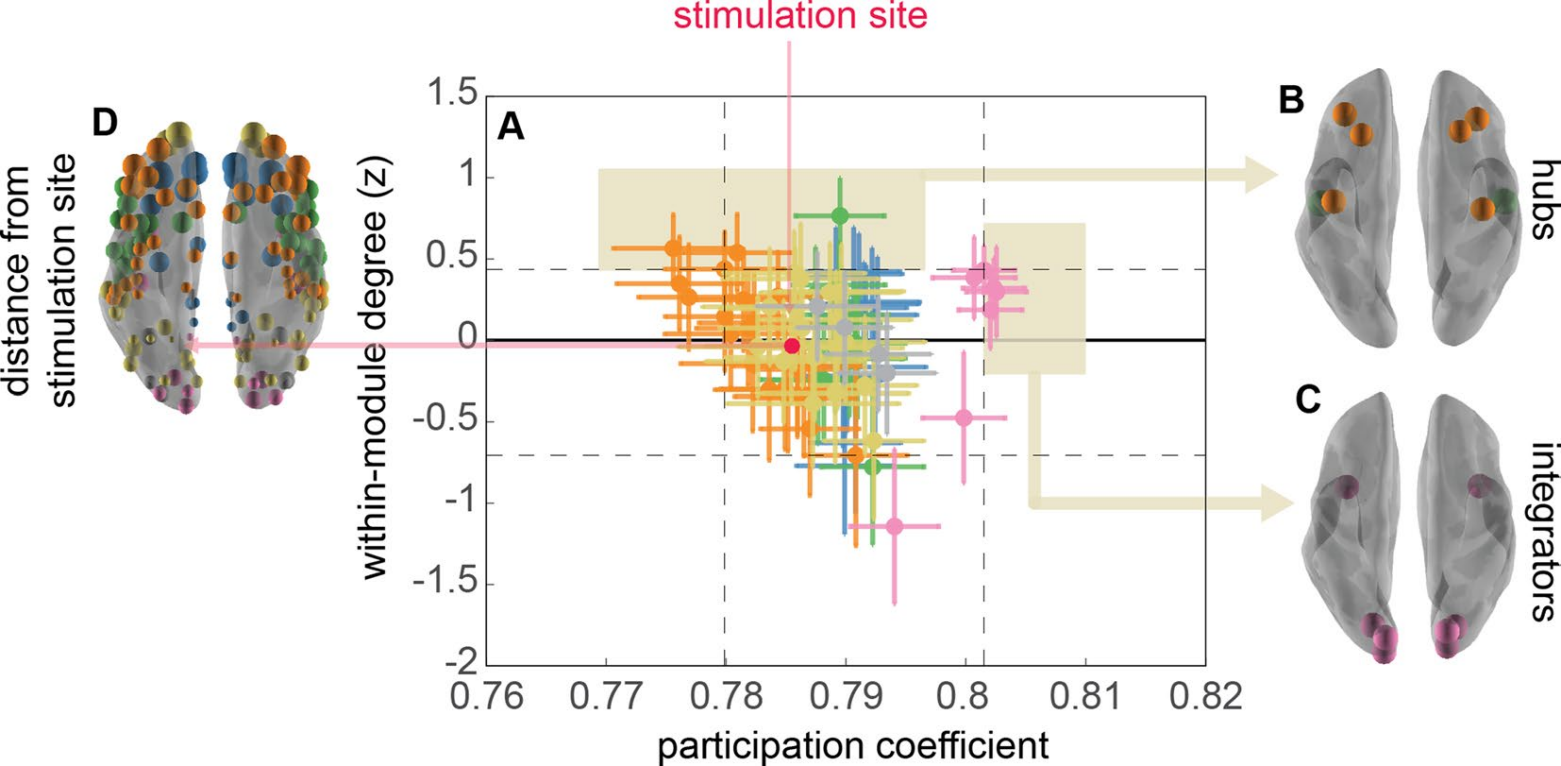
Characterizing the stimulation site. (**A**) Scatterplot of within-module degree and participation coefficients. Vertical dotted lines mark the central 90% of the participation coefficient across the brain (~ 133 nodes), and horizontal lines mark the central 90% of the within-module degree across all nodes all possible 148 regions. (**B**) Orbs plotted at the centroid of the regions that show the 95th percentile of the within-module degree, designating the most ‘hub-like’ nodes within the brain. (**C**) Orbs plotted at the centroid of the regions that show the 95th percentile of the participation coefficient, representing the ‘integrating’ nodes within the brain. (**D**) Orbs plotted at the centroid of all regions of the parcellated Destriuex atlas, where the size of node is scaled by the distance from the stimulation site or from the homologous region in the opposite hemisphere*.* The stimulation site (left intraparietal sulcus) is organized within the larger parietal, ventral temporal and orbitofrontal community (PT; shown in yellow).

An important consideration in network dynamics is whether individual nodes have a specialized role in the functional architecture, either in stabilizing the system or promoting information spreading across systems^16,37,48^. To determine whether the stimulation site, left intraparietal cortex, has unique structural properties in the system (i.e. high degree node, hub, etc.), we compared within-module degree, which is indicative of hub-like properties, and participation coefficient, a metric of integration, of the stimulation site to the overall distribution observed within our network (Fig. 5A). These metrics, unlike those previously discussed, are not dynamic. Instead, they characterize the strength and distribution of connectivity, as a static system, to the larger network as a whole.

Nodes with the highest within-module degree scores in this functional architecture were observed in dorsolateral prefrontal areas (orange; Fig. 5B); whereas regions with the highest participation coefficients during the visual tracking were in the occipital regions (pink; Fig. 5C). Nodes beneath the stimulation site in left IPS are neither hubs nor integrators, with a normalized node degree (− 0.01) and participation coefficient (0.78), a characteristic of many regions. Consequently, the community structure changes we observe following inhibitory rTMS over left IPS reflects the general susceptibility of the attention network to insult rather than unique connectedness of the targeted node.

### Behavioral results

**Figure 6:**
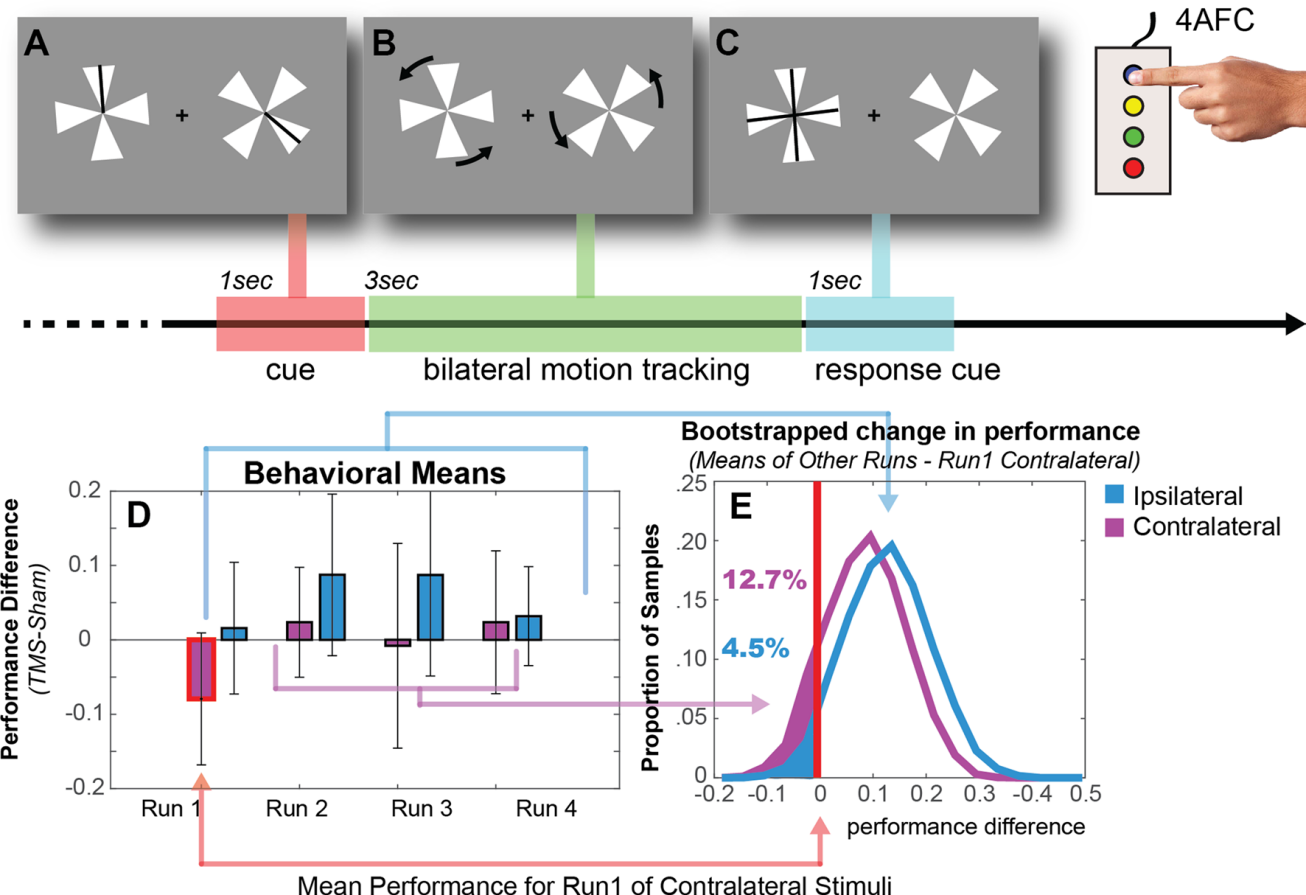
Experimental task. Participants (**A**) were cued to attend to one of the four pinwheel wedges in each hemifield, (**B**) then tracked those wedges through 3 s of rotation at a speed individually calibrated for 85% accuracy (3 up, 1 down staircase procedure, conducted prior to stimulation). One pinwheel terminated in an upright position and (**C**) participants indicated which of the four wedges (up, down, left or right) corresponded to the cued wedge. (**D**) The mean changes in tracking accuracy following rTMS (as compared to sham baseline) for the contralateral and ipsilateral hemifields. The impact of rTMS on behavior was most apparent in the first experimental run of tracking, contralateral to the stimulation site (in the right visual field). Error bars indicate SE mean difference (TMS-Sham). (**E**) A bootstrap analysis in which run and hemifield labels were discarded to generate null distributions of expected change in performance (10,000 iterations) shows only 12.7% and 4.5% of contralateral and ipsilateral scores, respectively, have larger effect size than the impact of rTMS on contralateral tracking in the first experimental run (~ 12 min).

Another important consideration is the impact of rTMS on behavioral performance in the motion tracking task. Participants, on average, exhibited an 8% decline in contralateral tracking for the first 12 min following rTMS, with performance returning to that observed following sham for the 24 min following (Fig. 6). Immediately following stimulation, the impact of rTMS on performance trended, but did not reach, statistical significance (t(6) = −1.31, *p* = 0.24), likely due to the relatively few numbers of tracking trials completed during each scan. There was no effect of the rTMS in the 24–48 min following the stimulation (Run 2: t(6) = −0.76, *p* = 0.47; Run 3: t(6) = −0.75, *p* = 0.48; Run 4: t(6) = −0.13, *p* = 0.90**).** Bootstrap analyses confirmed that tracking in the contralateral visual field immediately following stimulation scored within the lowest quartile of performance as compared to later timepoints and tracking in the ipsilateral visual field.

## Discussion

The goal of this study was to characterize the dynamics of mesoscale network architecture for visual attention disrupted by noninvasive neuromodulation. Inhibitory rTMS over left parietal cortex targets cortical networks that competitive mechanisms that support one’s ability to engage in bilateral visual tracking^20,49,50^. We observed rTMS to introduce transient downstream changes to community dynamics that outlasted the 15-min stimulation interval, predominantly within lateral prefrontal brain systems. Prefrontal nodes increased the probability of allegiance with nodes in the same system (recruitment) and to other systems (integration), integrating with many of communities at a high rate (high promiscuity). The changes in community dynamics following rTMS returned to the expected levels (as observed in a sham condition) within approximately 15 min. Together, these findings demonstrate intraparietal rTMS to destabilize the network architecture of nodes functionally connected to the stimulation site, inducing a temporary state of increased integration within an otherwise segregated system.

### Relationship to models of attention dysfunction (neglect and extinction)

That inhibition targeting the parietal nodes of the attention network would impact functional systems in lateral prefrontal cortex is not surprising in the context of the larger attention system, which spans dual frontoparietal pathways^1,2^ and couples with a frontal cognitive control system depending on task demands^51–54^. Moreover, results suggest that structural and functional connectivity of the entire frontoparietal system is critical for effective deployment of visuospatial attention^10,13,55,56^.

The intraparietal sulcus, the region targeted in this study, is an integral parietal node in the dorsal attention network. In previous studies, the delivery of inhibitory rTMS over left parietal cortex impairs one’s ability to engage in bilateral visual tracking^20^, a task sensitive to the sustained attention deficits observed in right parietal stroke patients^19,57–60^. The dorsal attention system is a neural system well-characterized by interhemispheric inhibitory connections that are more susceptible to excess excitation contralateral to a unilateral insult^61^. Neglect as a consequence of damage to either the frontal or parietal nodes in this network is attributed to a chronic imbalance in interhemispheric excitation and inhibition in the lesioned and contralateral parietal cortices, which can be re-established by delivering inhibitory rTMS to the healthy hemisphere^49,50,62^.

In terms of its network properties, the dorsal attention system lacks high centrality and instead is characterized strong homotopic transcollosal connectivity that shapes interactions between by contralateral maps of spatial attention^2,55^. These systems in particular are amenable to inhibitory noninvasive brain stimulation as a means for re-establishing normalized circuitry^28^. This is in contrast to nodes in the ventral attention network, which are characterized by high centrality, indicating they serve as hubs at which multiple brain networks are integrated^38,63^. As a result, direct insult to these regions are predicted to be induce more pervasive and severe impairments^16^. Our results also implicate dysfunction in the integrative function of frontal circuits that be a consequence of localized lesion to parietal cortex. By characterizing modular reorganization in the neuromodulated state, we gain insights into maladaptive reorganization patients exhibit in the chronic phase of stroke recovery.

### Comparison to theta-burst inhibitory neuromodulation

To our knowledge, this is the first characterization of large-scale network community dynamics following rTMS. Our findings, however, dovetail other reports of inhibitory neuromodulation as a means to disrupt global connectivity. Both rTMS and inhibitory “theta burst” stimulation (TBS) induce plasticity in local and distally connected circuits^27^. Network architecture indicates inhibitory TBS functionally isolates the stimulation site, decreasing local and long-range network connectivity when delivered over motor cortex, as assessed by evaluating the estimated resting state participation coefficient 25 min following stimulation^64^. The impact is also not local, as demonstrated by decreased resting state connectivity both within the stimulated hemisphere and in the unstimulated hemisphere in the alpha band following inhibitory TBS^65^. When applied using a multi-day accelerated protocol, TBS over left dorsolateral prefrontal cortex induces prolonged disruptions in network integration distributed throughout cortex, apparent 3 days after a week of stimulation^66^. Thus, neuromodulation has the potential to introduce sustained reorganization in large-scales systems beyond the targeted stimulation site.

### A comment on resting state versus task-based susceptibility

We found that the robust consensus community did not differ significantly between the rTMS and sham manipulations, with no significant change to nodal dynamics when evaluated over the entire scanning session. Only when considered via a sliding window within the brief interval directly following rTMS did we observe significant changes in nodal promiscuity, integration and recruitment. That network architecture measured over extended durations exceeding that anticipated for the effects of offline rTMS would be resilient to perturbation is not particularly surprising. This is also consistent with previous findings that noninvasive brain stimulation have no impact on global metrics of network modularity or clustering coefficients, even when measured in the resting state^65, 66^.

The robust community structure observed is also perhaps not entirely surprising in the context of previous work demonstrating the potency of tasks to increase connectivity between otherwise loosely connected networks. We note that, as compared to the resting state, task states are associated with increased network integration and decreased segregation of communities across a wide range of sensory, motor and cognitive tasks^37,67,68^, particularly for high cognitively demanding tasks^69,70^. For example, extended practice in figure sequences that increases motor automaticity also decreases the modularity of brain systems, while increases the number of transitions between network structures (i.e. the flexibility of the network)^72^. Moreover, those individuals that exhibit more flexibility of the network tend to demonstrate more learning. Thus tasks can have profound influence on the structure and neural dynamics of the underlying cortical architectures.

One hypothesis is that the increase in network integration during task-driven neural states reflects the strengthening of otherwise weak long-range connections, which in turn streamlines efficiencies while reducing neural flexibility into future brain states^71^. Our results are consistent with this proposal. Among the communities observed during visual tracking, some exhibited extreme dynamics compared to the population as a whole, as estimated using a null model. Nodes in the hetermodal parietotemporal cortex, which included the stimulation site, were among the most flexible, tending to coherently integrate with other communities, consistent with previous models of network ablation that reveal higher levels of network flexibility driven by hetermodal cortex^72^.

### Susceptibility of the intraparietal sulcus to neuromodulation

Some nodes have specialized roles within the network, determined by their connectivity both within and between communities^73^. Network hubs, which have many connections within their own community (high within-network node degree) but relatively low integration across networks (participation coefficients), have a stabilizing role in brain networks^37^. In contrast, integrator nodes form bridges across communities, serving as connectors across otherwise segregated cortical networks^74^. The balance between hubs and integrators facilitates the cooperation between the global synchrony and local computation, a hallmark of small-world networks and facilitating rapid communication between distal sights^75^. Whereas too much integration may promote disease spreading, not enough may diminish interaction between modules^39,76^.

Most relevant to our findings, network hubs and integrators have differential sensitivity to perturbation. Network hubs are more resilient to insult as compared to more peripheral nodes^48^, and the extent to which a node serves as a bridge is linked to more disease spreading, greater diaschisis, and more significant behavioral deficits^16,77^. In computational models, structural and functional connectivity is particularly susceptible to lesions impacting nodes of high centrality^38^. In situ lesion analyses bear the same findings and moreover show that damage to regions with high participation coefficients is associated with more significant behavioral impairments^17^. The impact on global network architecture is apparent, with damage to connector nodes increasing granularity of the community structure, quantified as increased modularity, in both the lesioned and healthy hemispheres^36^.

These findings support the proposal that neuromodulation will have the greatest clinical translation when delivered over high participation connector nodes with strong connectivity to nodes outside the network^78^. When impacted by acute brain injury, the left IPS (the targeted node in this study), is associated with disruptions to transcallosal homotopic connectivity, and disruptions to the functional balance of excitation and inhibition across hemispheres which, in turn is correlated to severity of hemispatial neglect following stroke^10,79^. The ventral attention network, on the other hand, is more closely associated with association fibers that support long-range connectivity within the same hemisphere^14^, that, and when disrupted, nonetheless have significant potential to alter network architecture^38,63^. Although dorsal parietal cortex has high scores of integration in the resting state^74^, the integrative properties of this region will vary dynamically over time and as a function of tasks^68^. Attention tasks, in particular, have the effect of increasing the participation coefficients across the brain overall as compared to rest.

In our measures, which included BOLD measures corresponding to intervals of attentive tracking and inter-trial rest, the left IPS had modal scores for node degree and participation coefficient as compared to the population, qualifying neither as a connector nor as a hub. Nonetheless, we observed the impact of the rTMS to destabilize the dynamics in communities downstream from the stimulation site. We therefore conclude that in the context of an attentive tracking task, the IPS behaves sufficiently as an integrator to facilitate the disruptive influence of rTMS through frontoparietal circuits.

### Conclusion

Inhibitory rTMS during visual tracking temporarily increases the within and between system integrative properties of functional systems of an otherwise stabilized and highly cohesive network. Rather than being restricted to the stimulation site, the influence of inhibitory rTMS on network architecture is most robust in the dynamics of nodal motifs outside of the targeted brain system. These findings suggest that connectivity and integrative properties of brain systems downstream from parietal cortex may underlie chronic attention dysfunction in parietal stroke.

## Materials and methods

### Participants

Nine healthy participants (mean age ± SD 27.72 ± 5.99 years, 7 males) participated in the experiment. Two participants were excluded from analysis due to gradient artifacts in the MR data incompatible with timeseries analyses. All participants had normal or corrected-to-normal vision. All participants met the inclusion criteria for participation in both TMS^80^ and MRI, and experiments were carried out in accordance with the guidelines set out for this method^80^. Participants provided written informed consent in accordance with and the experimental protocols were approved by the Institutional Review Board of the Beth Israel Deaconess Medical Center, Boston, MA .

### Procedure

Experimental procedures are detailed in Plow et al.^20^ and follow previous research^19,81^. Briefly, participants engaged in a bilateral visual tracking task in which they monitored 3 s of rotation by two rotating pinwheels positioned in the left and right hemifields (Fig. 6). Rotation speed was fixed at the rate that elicited 85% tracking accuracy across both the right and left visual fields, calibrated individually in a session prior to the rTMS/imaging. Participants completed 36 tracking trials per scan (18 per hemifield), each separated by a 20 s intertrial interval. Stimuli were generated in MATLAB using the Psychophysics Toolbox^82,83^.

Participants received 15 min of 1 Hz rTMS at 75% of the maximum stimulator output or sham, conducted on two experimental sessions in a counter-balanced order. Stimulation was applied using a MagStim device (MagStim, Whitland, Wales, UK) with a 70-mm figure-of-eight coil with the coil held such that the handle pointing posteriorly at an angle of 45° to the inter-hemispheric fissure, at an orientation that aligned it perpendicular to the left IPS. Sham stimulation was delivered identical to the TMS, with the exception that the coil was positioned with the edge at an angle perpendicular to the head. Stimulation was targeted to the left intraparietal sulcus (IPS), identified using frameless stereotaxic neuronavigation (BrainsightTM, Rogue Research Inc., Montreal, QC, Canada) co-registered with the individual participant’s anatomical images (average Talairach (mean ± SD): X = −23.4 ± 5.2, Y = −67.6 ± 4.3, Z = 52.9 ± 2.5).

fMRI data collection was initiated within 4 min following active and sham rTMS. fMRI data was acquired using a whole-body 3T Phillips scanner equipped with a standard birdcage headcoil. High-resolution (1 × 1 × 1.2 mm) T1-weighted MPRAGE images were subjected to automated surface-based segmentation and Desitrieux atlas parcellation^84^ in Freesurfer^85^. Functional images (EPI, TR = 2 s, 20 axial slices acquired interleaved, 2.4 × 2.4 × 4 mm with a 0.5 mm gap, TE = 55 ms, flip angle = 90°) were collected for three successive 12:12 min scans (366 volumes per scan).

Preprocessing of the functional scans was conducted in Brain Voyager (Brain Innovations), including correction for slice acquisition timing, removing linear trends, correction for rigid body movement within and across the volumes, and spatial smoothing (3 mm FWHM). fMRI volumes were then coregistered to the individual participant anatomies and morphed into surface space. The voxel timeseries for each surface region of interest was extracted, z-scored, then averaged into a single timeseries. This resulted in 148 timeseries corresponding to the mean brain activity from the 74 bilateral regions of the Desitrieux atlas.

### Network analysis

**Figure 7:**
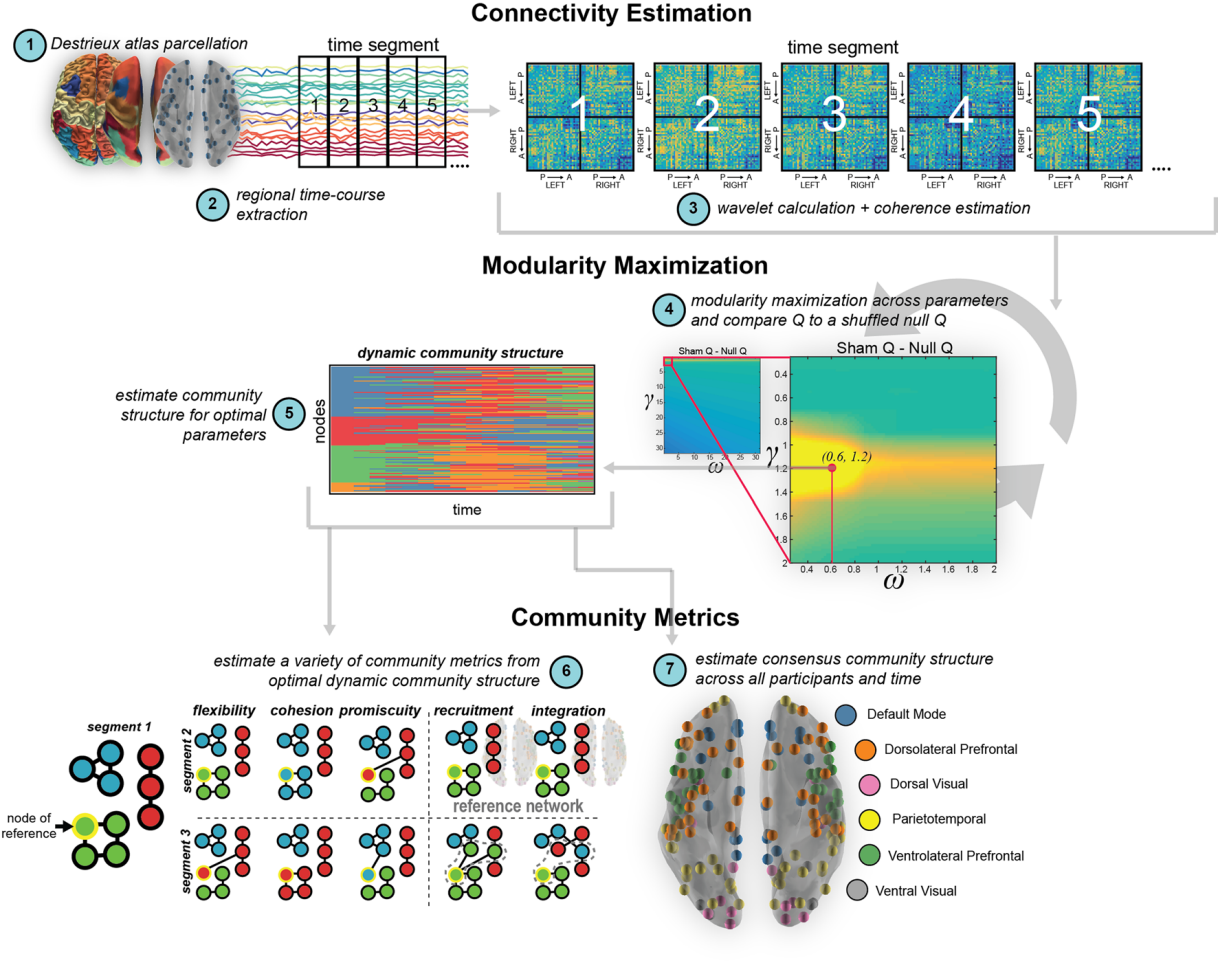
Dynamic community detection overview. Schematic of the approximate 7 steps completed for the dynamic community detection and metric estimation within this dataset. Briefly, (1) cortical regions of interest were parceled using the Destrieux atlas and (2) average regional time-courses were extracted from each of the 148 regions for each participant. (3) Timecourses were then passed through a continuous wavelet transformation and coherence was estimated between each pair of regions. (4) These connectivity matrices were then subjected to dynamic community detection across a set of parameters to determine the optimal “scale” in the dynamic community architecture. (5) A final community affiliation was calculated for the ‘optimal’ parameters, and these temporal labels were then used to (6) estimate community metrics (e.g., flexibility, cohesion, promiscuity, recruitment, and integration) and a representative community structure across participants (7).

Our analytical pipeline is outlined in Fig. 7 and follows procedures recommended in a review of community distillation of neural networks^86^. This procedure is also consistent with a previous investigation of single-pulse TMS network dynamics within band-specific intrinsic oscillatory activity^87^.

*Functional connectivity analysis* To estimate functional connectivity of brain networks, we used an undirected coherence measurement as computed between each pair of the 148 Desitrieux brain regions (Fig. 7, steps 1–3; for review, see^88^). The continuous wavelet transformation and coherence estimation was calculated using Matlab (Mathworks, Inc.) and a freely available wavelet toolbox^89^. Coherence was calculated across discrete non-overlapping 20 volume (40 s) temporal windows that spanned the 3 concatenated experimental runs (1,098 volumes) and averaged across frequencies corresponding to the task-relevant spectral content in fMRI BOLD connectivity, approximately 0.06–0.12 Hz^90–92^. This calculation yielded 54 unique coherence matrices representing the strength of each pairwise connection across the atlas parcellation.

*Dynamic community detection* To capture changes in networks over the course of the task, we utilized a multilayer dynamic community detection analysis^42,43^. Following our previous work on fMRI data^86,93^, we investigated the network effects of stimulation across the 54 time windows. The community detection algorithm optimizes a modularity quality function, Q, using a Louvain-like greedy algorithm^94^ to assign brain regions to communities. The community assignments are dependent on two parameters: (1) a structural resolution γ parameter (γ = 0.25 to γ = 31.62, in 20 logarithmically spaced steps) and (2) a temporal resolution ω parameter (ω = 0.25 to ω = 31.62, also in 20 logarithmically spaced steps). We search this parameter space to find the optimal parameter pair for this unique stimulation dataset, determined by comparing the mean value of optimized value Q in the experimental data to the mean value of Q in a shuffled ‘null’ model of the data.

Since we were most interested in the impact of rTMS on *normal* community structure, we used the sham condition for the parameter search and compared these estimates to those generated by a “null model” created by randomly shuffling the pair-wise coherence estimates and therefore destroying the correlational structure. This allowed us to draw conclusions of the effects of rTMS unbiased to the scale determined by the parameters. For each parameter pairing, we compared the observed model’s *Sham Q* and the null model’s *Null Q* (shuffled connectivity patterns) for each participant (Fig. 7, step 4). These parameters were optimized for γ = 0.6952 and ω = 1.1965, which were used in the reported analyses (Fig. 7, step 5).

Community detection was completed 100 times for each participant and each condition, as the community detection algorithm is non-deterministic^95^. This yielded 100 sets of community labels for the 148 nodes for each condition and for each of the seven participants across time. These iterations and final community labels were then used to calculate the subsequent metrics.

### Community metrics

We characterized the dynamic reconfiguration of spatially distributed regions following sham stimulation or active rTMS using metrics that can be broadly characterized within three categories:

*Individual node metrics* Using metrics from network science designed to understand the temporal dynamics of network changes, we quantify the movement of individual nodes between communities to determine the extent to which the community architecture is stationary or flexible. Node *flexibility, cohesion* and *promiscuity* are three metrics that have previously proven fruitful in describing how networks change following external stimulation^96,97^.

The *flexibility* of each node corresponds to the number of instances in which a node changes community affiliation, *g,* normalized by the total possible number of changes that could occur across the layers *L*^42^. In other words, the flexibility of a single node *i*, *ξ*_*i*_, may be estimated with

1 $$\xi_{i} = \frac{{g_{i} }}{L - 1},$$

where L is the total number of temporal windows.

*Cohesion* quantifies the extent to which nodes shift community affiliations *jointly* across time, or the movement of network motifs^98^. Node cohesion is derived from a pairwise measurement, cohesion matrix *M*, where edge weight *M*_ij_ denotes the number of times a pair of nodes moves to the same community together divided by L − 1 possible changes. Thus, the cohesion strength of node *i*, *Ω*_i_, as used in this work, is then defined as follows.

2 $${\Omega }_{i} = \mathop \sum \limits_{j \ne i} M_{ij} .$$

*Promiscuity* characterizes the fraction of all the communities in the network in which a node participates at least once^96^. Importantly, this metric may be used to determine whether a node’s flexibility is high simply because it is switching between two communities or across all communities.

Taken together these individual node metrics are used to distill the network dynamics of each node, estimating how much a node changes across time but lacking some sensitivity to *how* the node changes across time. The following metrics of nodal allegiance inform on the latter.

*Dynamic allegiance metrics* The second class of metrics quantifies the dynamics in node allegiance, the probability that any two nodes co-exist within the same community, as induced by rTMS. These metrics quantify the rapid reconfiguration within- and between-system dynamics of nodal motifs, with the comparison architecture established by a representative *brain system*^46,99^. In this study, the architecture of the reference brain system is constrained by the visual tracking task, and these metrics allow us to determine dynamic changes induced by rTMS of motifs within that predefined system.

*Consensus community structure* To estimate the most representative community structure, we follow the methodology determined by Doron et al.^100^, modified to estimate the most representative community structure for each individual across time. This scheme utilizes the z-score of the Rand coefficient^101^ which compares each pair of communities within the temporal dataset in terms of the total number of pairs that are in the same community. After each partition is compared, the structure with the highest Rand z-score is the most representative community. We compute this representative community from the Sham condition, which reflects system architecture during visual tracking but without the stimulation intervention. This process is similarly completed across participants, where the consensus community is built on each individual’s representative community (Fig. 7), providing the common community structure across our participant group and time.

We next estimated two metrics, *recruitment* and *integration*, which describe whether the node changes affiliation within its own brain system (recruitment) or with other systems (integration). Mathematically, both metrics begin by first estimating *module allegiance*. Similar to the cohesion matrix, we define allegiance matrix *P*, where edge weight *P*_ij_ denotes the number of times a pair of nodes moves to the same community together divided by L − 1 possible changes. Then, both measurements are found by averaging the module allegiance within its brain system or outside its brain system. For this estimate, we call the aforementioned representative community as being a group of nodes *C* such that *C* = {*C*_1_…*C*_*k*_} where k is the number of communities in the representative community structure. *Recruitment* is the average probability over the dynamic window over which a region retains the same community affiliation as nodes from its own community in the reference system (*k*_1_ = *k*_2_). *Integration* is the average probability that a region is in other communities other than it’s own (k_1_ ≠ k_2_). In other words, recruitment is the average *allegiance* within its own community (as defined by the reference community) where integration is the average *allegiance* with every other community.

*Metric changes across time *Dynamic changes to node and community metrics were also estimated across time for each participant and iteration of the community algorithm (Fig. 7, Step 5). The timeseries were epoched into sets of size 10 units here (10 community windows = 200 volumes), and all metrics were estimated from this epoch. To estimate time-evolving metric changes, the epoch was then consecutively shifted by 1 window (20 volumes) until all of the 54 community windows were used in the time evolving metric estimation (Fig. 3).

*Characterizing the stimulation site* To characterize the connectivity structure of the node underlying the stimulation site, we also computed more common network metrics *within-module degree* and *participation coefficient*^75,102^ using the average pairwise coherence estimate from the Sham condition and the Matlab Network Community Toolbox (https://commdetect.weebly.com/). Similar to the previously defined recruitment and integration metrics, these metrics also require the definition of a reference community structure. Here, we used the same consensus community across participants (Fig. 1) and then estimated degree and participation relative to this structure.

*Within-module degree* is defined as the average coherence between a node and other nodes in the same community, z-scored to normalize across the community population. *Participation coefficient* denotes the diversity of a node's connectivity, computed as the proportion of connections that are accounted for by a node’s own community rather than to nodes in communities other than its own. Thus, a participation coefficient of 0 denotes that the connectivity is restricted to one’s own community and a value of 1 denotes connectivity restricted to every other community besides its own.

We use these measurements to determine whether, in a classical sense, the stimulated node is an integrator or a hub. Where previous research has used a strict limitation on node degree (z > 2.5) and participation coefficient to define hubs and classify nodes, we use a soft classification of *hubs* and *integrators*, loosely defining both based on where these nodes lie in the distribution of nodes within our dataset (> 95% of other nodes). We note that although overwhelming previous research evaluates hubs and integrators in the resting state, this analysis was conducted on task-driven community structure, and we know that fMRI BOLD measurements during a task show fundamentally different network configurations^67^. Thus, the hubs and integrators shown in Fig. 5 should only be interpreted within the confines of this study’s task.

### Statistical significance

*Active versus sham TMS, metric comparison* A series of paired-sample t-tests were performed to compare the derived flexibility, cohesion, and promiscuity metrics over the entire 36 min period following rTMS (Fig. 2) to the metrics derived from the sham condition. This was completed for each of the 148 nodes of the brain. Due to the high volume of statistical tests, the False Discovery Rate (FDR) was used to correct for multiple comparisons. A q = 0.05 was used as the statistical cut-off^44^.

*Active versus sham TMS, correlations* Due to the highly similar metrics across both conditions over the entire temporal interval (36 min), we also estimated the mutual relationship between the estimated mean across subjects for the active and sham rTMS conditions using a correlation analysis. For each metric, the observed mean value across subjects of the 148 nodes was compared in the both conditions. Reported *r* and *p* values indicate the strength and significance of this relationship.

*Population distributions of node metrics* As a follow-up statistical test to the unobserved differences between the active rTMS and sham conditions, we used a Monte Carlo method to identify the nodes (and networks) with the most extreme dynamics across both conditions. Probability distributions were derived from 10,000 random drawings of the estimated metrics across subjects and nodes for all conditions, separately for flexibility, cohesion, and promiscuity. For each of the 10,000 iterations, the metrics (e.g., flexibility) across the 7 participants and 148 total regions were combined to create a single vector of scores. A random 7 values were drawn from this vector and averaged to create a single mean estimated group score from the unlabeled distribution. Thus, the derived probability distribution is representative of the grand mean across subjects and nodes and may be used to indicate which observed metrics would not be observed due to random draws of unlabeled data.

*Time-evolving metric comparison* To test the hypothesis that the unobserved differences were due to the decay of the active stimulation effects, we computed change in network dynamics between the active and sham rTMS using sliding time-evolving windows (1 window = 200 volumes). Differences in the metric scores across time and the brain regions was assessed using the average of a sum of squared differences across nodes of the brain. This provides a coarse estimate of *any* observed change due to the rTMS. For each time point within the 10 min following rTMS (a time equal to the length of stimulation), we compared the subject’s SSD estimate across nodes to zero using a single sample t-test. Due to the quantity of tests completed across time, the observed *p* values were subjected to a correction for multiple comparisons using the FDR correction. Corrected and uncorrected time points are shown in Fig. 3.

*Behavioral changes* Observed behavioral changes indicated a decrease in the mean performance within the first run of scanning following rTMS of the stimulus contralateral to the stimulation site. Due to the small sample size (N = 7, 18 trials per condition in the first 12 min following stimulation) and the unequal sample means of behavior (4 runs for ipsilateral performance vs 3 runs for contralateral performance), this single mean of contralateral performance difference (rTMS-Sham performance) was compared to a probability distribution of bootstrapped means using a Monte Carlo method. The distributions of behavioral performance were estimated by computing the difference between the mean contralateral performance in Run 1 and against seven values randomly drawn from one of two vectors of the estimated performance from the other runs (i.e., ipsilateral performance in the 4 runs and the 3 runs of contralateral performance). This was repeated for 10,000 random draws each for ipsilateral and contralateral performance. The derived probability distribution is representative of the grand mean across all subjects and stimulus types (contralateral/ipsilateral to the stimulation site) and may be used to indicate significance of the observed contralateral performance in the first run (compared to all others). A one-tailed test of the effect of rTMS was computed by counting the proportion of the 10,000 differences that were below zero.
